# Emerging role of neutrophil extracellular traps in the complications of diabetes mellitus

**DOI:** 10.3389/fmed.2022.995993

**Published:** 2022-08-23

**Authors:** Areez Shafqat, Saleha Abdul Rab, Osama Ammar, Sulaiman Al Salameh, Anas Alkhudairi, Junaid Kashir, Khaled Alkattan, Ahmed Yaqinuddin

**Affiliations:** ^1^College of Medicine, Alfaisal University, Riyadh, Saudi Arabia; ^2^Center of Comparative Medicine, King Faisal Specialist Hospital and Research Centre, Riyadh, Saudi Arabia

**Keywords:** diabetes mellitus, neutrophil extracellular traps, atherosclerosis, thrombosis, macrovascular and microvascular complications

## Abstract

Immune dysfunction is widely regarded as one of the central tenants underpinning the pathophysiology of diabetes mellitus (DM) and its complications. When discussing immunity, the role of neutrophils must be accounted for: neutrophils are the most abundant of the circulating immune cells and are the first to be recruited to sites of inflammation, where they contribute to host defense *via* phagocytosis, degranulation, and extrusion of neutrophil extracellular traps (NETs). NETs are composed of DNA associated with nuclear and cytosolic neutrophil proteins. Although originally reported as an antimicrobial strategy to prevent microbial dissemination, a growing body of evidence has implicated NETs in the pathophysiology of various autoimmune and metabolic disorders. In these disorders, NETs propagate a pathologic inflammatory response with consequent tissue injury and thrombosis. Many diabetic complications—such as stroke, retinopathy, impaired wound healing, and coronary artery disease—involve these mechanisms. Therefore, in this review, we discuss laboratory and clinical data informing our understanding of the role of NETs in the development of these complications. NET markers, including myeloperoxidase, citrullinated histone H3, neutrophil elastase, and cell-free double-stranded DNA, can easily be measured in serum or be detected *via* immunohistochemical/immunocytochemical staining of tissue specimens. Therefore, NET constituents potentially constitute reliable biomarkers for use in the management of diabetic patients. However, no NET-targeting drug is currently approved for the treatment of diabetic complications; a candidate drug will require the outcomes of well-designed, robust clinical trials assessing whether NET inhibition can benefit patients in terms of morbidity, quality of life, health expenditures, and mortality. Therefore, much work remains to be done in translating these encouraging pieces of data into clinical trials for NET-targeting medications to be used in the clinic.

## Introduction

Diabetes mellitus (DM)—both type 1 (T1DM) and type 2 (T2DM)—is characterized by hyperglycemia, which chronically leads to several vascular complications. The global prevalence of DM is estimated to be 9.3% (463 million people) and is projected to rise to 10.9% (700 million people) by 2045. The healthcare and economic burden of DM is proportionately massive: the 10th edition Atlas of the International Diabetes Federation estimates the number of deaths caused by diabetes in 2021 at 6.7 million.

Diabetes mellitus is a major cause of cardiovascular disease, including strokes and myocardial infarction (MI), and is the leading cause of non-traumatic limb amputations, end-stage renal disease (ESRD), and adult blindness (1). This makes diabetes mellitus a major threat to public health and a risk factor for premature death. To understand these complications better and provide a foundation for the development of interventions, it is essential to study the potential mediators of these effects. In this regard, autoimmune destruction of pancreatic β-cells is central to the development of T1DM (2). Similarly, although T2DM is underpinned by peripheral insulin resistance, it features chronic low-grade sterile inflammation in which the role of adaptive immunity has been extensively studied (3).

Neutrophils are important to acknowledge when studying inflammation; they are the most abundant of all immune cells and the first to be recruited to sites of acute inflammation. Neutrophils contribute to host defense by phagocytizing microbes and degranulating to release antimicrobial effectors. In 2004, Brinkmann et al. described a novel neutrophil function called NETosis, which involved the release of DNA decorated with cytosolic and granular proteins as web-like structures called neutrophil extracellular traps (NETs) (4).

The beneficial roles of NETs remain best characterized in the context of infections (5). Generally speaking, neutrophils release NETs when microbes overwhelm other neutrophil functions. For instance, neutrophils undergo NETosis in fungal infections, as fungi are too large for neutrophils to phagocytose (5, 6). This is underscored by studies in humans and mice, which show that defective NET production predisposes to severe and recurrent fungal infections (6, 7). This is not the case for bacteria, which can be phagocytized and eliminated *via* the respiratory burst. Therefore, NET-mediated protection for bacterial infections is more selective. For instance, Brinkmann et al.'s study and other reports have shown that NETs bind gram-positive and gram-negative bacteria, preventing their dissemination—which is essential in combating septic shock—accompanied by the degradation of virulence factors by NET-associated proteases (4, 8). Furthermore, interestingly, impaired killing of *Shigella flexneri and* Group A Streptococci is seen in NET-deficient mice, suggesting that perhaps NETs exert unique, non-redundant antibacterial functions in these infections (9). By contrast, data on NETs as mediators of protection in parasitic infections is inconclusive, whereas they are primarily considered pathologic in viral infections such as influenza and SARS-CoV-2 (5).

However, NET production is mainly inappropriate in other diseases, including autoimmune and metabolic disorders and cancers. Indeed, NET production is a significant driver of a whole host of non-infectious pathologies. In keeping with the focus of this review, NET markers—MPO-DNA, cit-H3, and NE—are elevated in diabetic mice and humans who develop these complications, thereby paving the way for clinical studies evaluating the robustness of NET markers as prognostic markers and therapeutic targets. Therefore, in this review, we survey the literature on the role of NETs in the complications of DM and suggest how future research can expand upon these findings.

## NETosis, NET formation, and methods of detection

**Figure 1:**
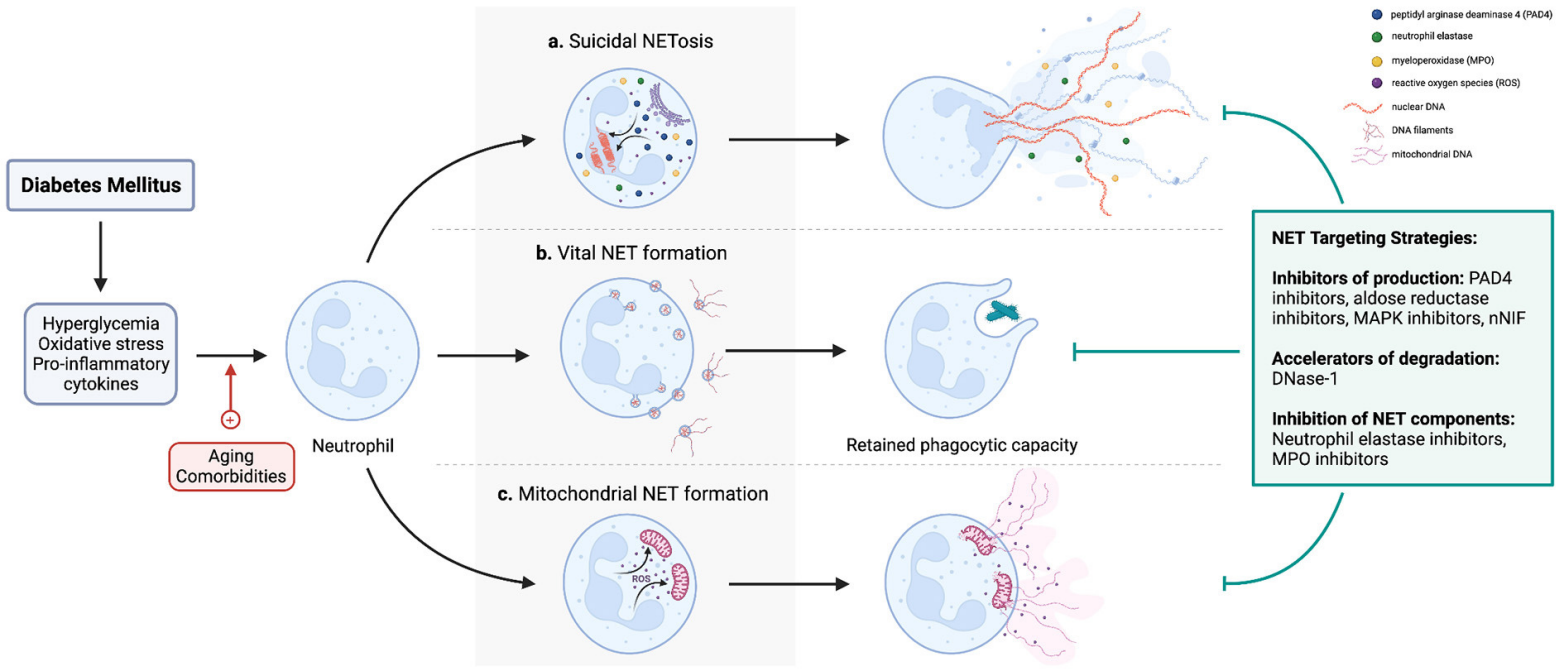
A diagrammatic representation of the different pathways of NETosis and NET production. Importantly, whereas (a) NETosis entails release of NETs with concomitant neutrophil apoptosis, neutrophils retain viability and effector functions after (b) vital and (c) mitochondrial NET production. In the context of diabetes, hyperglycemia activates NADPH oxidase, and oxidative stress and elevated pro-inflammatory cytokines consequent to a chronic sterile inflammatory state induce NETosis and NET production. However, if and how age and co-existing cormorbidities such as obesity contribute to these pathways are unclear. Since numerous studies have implicated NETs in the pathogenesis of diabetic complications, strategies to mitigate the effects of NETs include preventing their production (e.g., PAD4 inhibitor, NE inhibitors), accelerating their degradation (DNAse-1), or inhibiting NET components to ameliorate their respective pathologic effects (NE inhibitors, other protease inhibitors e.g., MMP-9). However, it should be noted that no drug currently targeting NETs is approved for prevention or treatment of diabetic complications.

NETosis is a unique form of cell death (suicidal NETosis) that produces NETs. Neutrophils can also produce NETs while still retaining membrane integrity and effector functions, *via* a mechanism called vital NET production. Another form of NETs contains largely mitochondrial DNA (mtDNA) and is termed mitochondrial NET production. Therefore, the term “NETosis” specifically refers to NET release accompanied by lytic neutrophil death and does not include vital and mitochondrial NET formation (Figure 1).

NETosis begins with disruptions to the characteristic lobular nuclear architecture of neutrophils and chromatin decondensation (10). This is followed by the disintegration of the nuclear membrane with the release of DNA and histones into the cytosol. Lastly, the plasma membrane ruptures occur, thereby extruding NETs into the extracellular space (5). From a mechanistic standpoint, these cellular pathways are predicated on the activation of NADPH oxidase-2 (NOX2), which produces reactive oxygen species (ROS) *via* a respiratory burst. It is important to note that protein kinase C (PKC) isoforms and mitogen-activated kinases (MAPK) regulate NOX-2 to influence NETosis and NET formation. ROS trigger the release of myeloperoxidase (MPO) and neutrophil elastase (NE) from azurophilic granules, both of which translocate to the nucleus and synergize to partially degrade histones and thereby loosen their association with DNA—resulting in chromatin decondensation (11, 12). Importantly, DNA inhibits proteases, meaning this close association between MPO, NE, and DNA allows both enzymes to retain their enzymatic activity in a highly proteolytic environment, which later enables them to exert protective or pathologic effects extracellularly. Chromatin decondensation is augmented by peptidylarginine deaminase 4 (PAD4)—also ROS-dependent—which citrullinates histones to decrease their positive charge, hence reducing their electrostatic attractions between DNA and loosen the chromatin (13, 14). This is followed by disassembly of the nuclear envelope and release of chromatin associated with histones, NE, and MPO into the cytosol. Finally, pores in the plasma membrane form, through which NETs are extruded into the extracellular space, resulting in NETosis (15).

Neutrophils can alternatively release NETs without lysing, a process termed vital NET formation. This allows neutrophils to retain their effector functions, such as chemotaxis, phagocytosis, and degranulation. Nonlytic NET formation has particularly been studied in the context of infection, where live neutrophils have been visualized as releasing NETs during crawling but without lysing (16, 17). Importantly, nonlytic NET production occurs independently of NADPH-oxidase. Mechanistically, lipopolysaccharides in the cell wall of gram-negative bacteria can activate platelets *via* Toll-like receptor 4 (TLR4). Activated platelets can adhere to neutrophils *via* P-selectin and stimulate NET production through platelet-derived high mobility group box protein B1 (HMBG1) binding to receptors for advanced glycation end-productions (RAGE) on neutrophils (18, 19). In vital NET production, activated PAD4—a step that occurs independently from NADPH oxidase—translocates to the nucleus to citrullinate histones, followed by extrusion of decondensed chromatin, histones, and other embedded protein in blebs of nuclear membrane, which is then resealed (20). Notably, Yousefi et al. reported ROS-dependent nonlytic NET production containing mtDNA and not nuclear DNA, termed mitochondrial NET formation (21). In terms of upstream regulators, activation of TLR4 and complement factor 5a (C5a) receptor after the application of GM-CSF result in mitochondrial NET formation (21).

Collectively, this is compelling evidence for the heterogeneity of NETs, depending on the type of the activating stimulus. Importantly, NETs heterogeneity could have important clinical implications as different stimuli may confer differential NET compositions. For instance, NETs containing mitochondrial DNA would not contain histones. How these varying compositions translate into differing protective and pathologic functions of NETs remains unclear. In the context of diabetic complications, studying the inducers of NETosis and NET production as will be discussed later herein may provide clues as to the types of NETs present in these circumstances. Numerous lines of evidence have shown NET production and NET markers to be elevated in DM patients. From a mechanistic perspective, hyperglycemia activates NOX2, evidenced by heightened ROS generation by neutrophils after high glucose stimulation, and its abrogation following treatment with NOX inhibitors (22). However, the interplay between or independent contributions of comorbidities and lifestyle habits that frequently accompany diabetes—such as obesity, hypertension, smoking, and aging—to NET production in addition to hyperglycemia remain unexplored. In this regard, whereas obesity has been reported to reduce neutrophil NET-producing capacity, all of the other aforementioned factors promote NET production or feature NET contributions in their pathophysiology (23–26). Therefore, in a clinical context, these factors may represent important variables to control for when assessing NETs in diabetics.

There is currently no gold standard modality for detecting NETs. For this reason, studies have advocated using combinations of detection methods rather than a single method (27). For instance, markers of NET production include MPO, citrullinated histone-3 (cit-H3), NE, and extracellular DNA, which have been measured in the serum of patients with inflammatory illnesses. Other techniques, such as flow cytometry, immunocytochemistry, and immunohistochemistry are also commonly used to detect NETs (28, 29). Investigators caution that DNA and histones are also released by necrosis and tissue death, underscoring the importance of objectively defining what constitutes NETs. In this regard, most studies identify NETs by colocalizing at least three NET components, most commonly, DNA, histones, NE, and/or MPO. Lastly, live cell imaging and intravital imaging may allow real-time observations of NETs *in vivo* (30, 31).

## Role of NETs in the complications of diabetes

The so-called macrovascular complications of diabetes are related to the development of atherosclerosis, chronically resulting in coronary artery disease (CAD), MI, strokes, and peripheral vascular disease. Microvascular complications of diabetes include retinopathy, nephropathy, and neuropathy. The following section summarizes and interprets basic science and clinical data associating NETs with the development and outcomes of the aforementioned complications.

### Coronary artery disease

Diabetes is associated with an increased risk of atherosclerosis, with coronary artery disease (CAD) remaining the most common cause of mortality in diabetics (32). CAD occurs secondary to the development of atherosclerotic plaques, which progressively occlude coronary circulation, causing stable angina. Acute coronary syndrome (ACS) is the major complication of CAD and is typically the result of the rupture of an atherosclerotic plaque, upon which a thrombus is formed. The pathogenesis of atherosclerosis begins with endothelial cell dysfunction, with resultant monocyte migration into the tunica intima, where the monocytes eventually transform into foam cells and drive plaque development. T-cells also comprise a major portion of the immune cell landscape of both intact and complicated atherosclerotic plaques. However, studies have also investigated (1) the presence of NETs in atheromas, (2) whether NETs contribute to plaque development and/or complications, and (3) if NET markers correlate with the severity of CAD or outcomes of ACS.

Megens et al. initially demonstrated the presence of NETs in both mice and human atherosclerotic plaques (33). Subsequent immunohistochemical staining for NETs in human atherosclerotic plaques showed them to be concentrated at sites of superficial erosions next to apoptotic endothelial cells, a process that results in thinning of the overlying fibrous cap making the plaque more prone to rupture and subsequent thrombosis (34). Histological analyses conducted on plaque specimen samples from the human carotid artery reveal neutrophils and NETs to be localized in plaques with a large lipid and low fibrous content, which are indicators of plaque instability (35). These results suggest that NETs play a role in plaque destabilization or complications. A recent study, attempting to explore the mechanisms underlying this observation, revealed that citrullinated histone H4 (cit-H4) kills smooth muscle cells and endothelial cells to cause fibrous cap thinning (36, 37).

Accordingly, immunohistochemical staining of human coronary artery atheromas reveals the presence of NETs in complicated plaques (including intraplaque hemorrhage, eroded plaques, or fibrous cap ruptures) but not in intact plaques (38). This is backed by clinical data suggesting that neutrophils may contribute to plaque complications: Borissoff et al. showed NET components—dsDNA, nucleosomes, cit-H4, and MPO-DNA complexes—to be significantly higher in patients with severe CAD than in healthy controls and to be associated with the severity of luminal stenosis and occurrence of major cardiac events, including ACS, percutaneous coronary intervention, coronary artery bypass grafting and cardiac death (39). NETs may also impair plaque resolution: Josefs et al. showed NETs impairing plaque resolution in mice by promoting macrophage-mediated plaque inflammation, evidenced by elevated levels of anaerobic glycolysis and inflammasome (40). Furthermore, NETs decline in resolving plaques, and treatment with exogenous DNAse-1 reduced atheroma NET content and macrophage inflammation, promoting plaque resolution when given adjunctively to lipid-lowering therapy (40).

However, despite these encouraging preclinical findings and intriguing hypotheses, the clinical utility of NET markers in diabetics at risk of CAD has not been proven. Conversely, a recent paper studying NET markers in T1DM and their link to CAD did not find any significant differences in NET markers between T1DM patients and age-matched healthy controls. Furthermore, NETs did not differ significantly in type 1 diabetics according to the presence of CAD.

### Myocardial infarction

Crucially, NETs are well-recognized mediators of thrombosis: NETs interact with platelets to promote thrombogenesis, NETs promote the generation of thrombin, and NET constituents such as tissue factor, factor XII, histones H3 and H4, cell-free DNA, and fibrinogen, are all prothrombotic (37). Therefore, researchers have investigated the contribution of NETs to coronary thrombosis and MI in depth.

Indeed, higher circulating NET markers (MPO-DNA, NE-DNA, cit-H3) and platelet activation markers (soluble P-selectin) confer an increased risk of major cardiovascular events (MACEs) 12 months after MI (41). NETs are found in coronary artery thrombi, particularly in fresh thrombi rather than older, organized thrombi (42). Another study used colocalization of NE and extracellular dsDNA to identify NETs in 25% of coronary stent thrombi (43). Importantly, Cui et al. reported higher dsDNA levels in ACS patients than in stable angina (SA) patients and healthy controls, informing the potential utility of dsDNA as a biomarker in this setting (44). Furthermore, in the ACS group, significant differences were shown in dsDNA levels observed between unstable angina (UA), non-ST elevation myocardial infarction (NSTEMI), and ST-elevation myocardial infarction (STEMI) patients (44). NET markers dsDNA, MPO-DNA, and cit-H3 reveal a higher NET burden in STEMI patients at the culprit site lesion as compared to other areas such as the femoral artery (45). Furthermore, NET burden at the culprit site correlates positively with infarct size, assessed by cardiac enzyme elevation and cardiac MRI, and left ventricular dysfunction, assessed by wall-motion score index at a year follow-up (45). Circulating MPO and NE levels significantly decrease after MI treatment (46).

Neutrophils also play a role in acute cardiac fibrosis post-MI, as necrosis is followed by inflammation and healing by fibrosis, but crucially release NETs that contribute to chronic cardiac fibrosis and remodeling after MI (47). Pathologic cardiac remodeling increases the risk of ventricular aneurysm formation, which may be lethal. In this regard, NETs are detected in ventricular aneurysms in both humans and mice. NETs are also elevated in the peripheral blood of patients that develop ventricular aneurysms (47).

Importantly, DNAse-1, an enzyme that degrades NETs, correlates negatively with infarct size and positively with ST-segment resolution, and *ex vivo* administration of DNase-1 accelerates lysis of coronary thrombi (45). In mouse models of MI, administering a PAD4 inhibitor GSK484 intraperitoneally reduces infarct size and improves cardiac function (48). DNAse-1 administration also abrogates NET-induced cardiac fibrosis both *in vivo* and *in vitro*, suggesting a prognostic and therapeutic role of NETs in pathologic cardiac fibrosis and secondary ventricular aneurysms (47).

### Stroke

Ischemic stroke is a major macrovascular complication of diabetes. Similar to CAD, the role of NETs in plaque destabilization and thrombosis is highly relevant in ischemic stroke. NETs indeed are detected in almost all thrombi retrieved from ischemic stroke patients analyzed by immunohistochemical staining (49). Hyperglycemia is associated with poor outcomes in ischemic stroke patients. To explain this, hyperglycemia induces NETosis; NET infiltration is more extensive in ischemic stroke thrombi retrieved from hyperglycemic patients compared to normoglycemic patients (50). Importantly, blocking NET formation with Cl-amidine (a PAD4 inhibitor) reduced brain infarction volume and alleviated neurologic deficits in diabetic and wild-type (WT) mice (50).

Interestingly, in contrast to coronary thrombi, NETs are predominantly present in older ischemic stroke thrombi rather than fresh thrombi (49). In murine models of ischemic strokes, dense neutrophilic infiltration and NETs are present throughout the brain tissue (51). Mechanistically, platelet-induced NET formation *via* high-mobility group box 1 protein (HMGB1) expression was found to be the dominant mechanism of NET formation in these models, exemplified by HMGB1-depleted mice showing significantly lower plasma NET levels after stroke with greatly improved clinical outcomes (51).

Importantly, NETs may increase resistance to tissue plasminogen activator (tPA) therapy; NET content cerebral thrombi correlated significantly with endovascular therapy procedure length and the number of thrombectomy device passes performed to achieve successful recanalization (52). Importantly, combining DNase-1 with conventional tPA more effectively lysed patient thrombi as compared to tPA alone (49, 52). However, DNase-1 alone was not effective in *ex vivo* clot lysis, which is in contrast to findings from coronary thrombi (52). To explain this, Farkas et al. compared NET content in ischemic stroke clots, coronary thrombi, and peripheral artery disease (53). NET content was lowest in ischemic stroke thrombi, perhaps explaining why DNAse-1 alone was unable to effectively degrade these thrombi. Other than accelerating NET degradation *via* DNAse-1, inhibiting NET formation by targeting PAD4 is a feasible strategy: PAD4 overexpression impairs revascularization and vascular remodeling in stroke, while PAD4 inhibition restores angiogenesis (54). Lastly, treating mice with neonatal NET-inhibitory factor (nNIF) reduced the size of brain infarcts, improved long-term neurological and motor function, and enhanced survival after stroke (51). Another crucial finding from this study was that nNIF improved stroke outcomes in diabetic mice, even when administered 1 h after stroke onset (51).

These findings warrant investigations into NETs as prognostic markers for stroke patients including diabetics. Furthermore, clinical trials evaluating a combination of DNAse-1 and tPA as stroke therapy vs. conventional tPA alone are needed to confirm the utility of NETs as therapeutic targets in stroke. The same applies to nNIF, which, as mentioned above, has recently shown encouraging results as a stroke therapy.

### Diabetic retinopathy

Diabetic retinopathy (DR) is the most common microvascular complication of diabetes, being more common in T1DM than T2DM, and is the leading cause of adult blindness globally (55, 56). A third of diabetic patients suffer from DR, of which a third is vision-threatening (57). DR is divided into two pathophysiologically distinct stages based on fundoscopic findings: an early non-proliferative (NPDR) stage and a later proliferative (PDR) stage. NPDR is characterized by pericyte and endothelial cell dysfunction with a consequent increase in capillary permeability and capillary occlusion, manifesting on fundoscopy as micro-aneurysms, hard exudates, and hemorrhage. NPDR is often clinically asymptomatic (58). PDR, on the other hand, represents an advanced stage, occurring secondary to angiogenesis mediated by vascular endothelial growth factor (VEGF). It manifests as neovascularization on fundoscopy but is also sometimes accompanied by vitreous hemorrhage and/or retinal detachment. PDR often results in pronounced visual impairment. More importantly however is diabetic macular edema (DME), which can occur at any stage of DR and is the most common cause of blindness in diabetic retinopathy (58).

Hyperglycemia is a major player in the pathogenesis of DR. The retinal blood vessels dilate in response to the high blood glucose, increasing local blood flow (59). Hyperglycemia also induces apoptosis of pericytes, increasing capillary permeability but also causing outpouching of these segments of the capillary wall to form micro-aneurysms. Apoptosis of endothelial cells also occurs, collectively leading to hypoxia and induction of hypoxia-inducible factor-1α (HIF-1α), the transcription factor which activates the expression of vascular endothelial growth factor (VEGF) (58).

Inflammation also contributes to the pathogenesis of DR. For instance, leukostasis is a central part of occlusion of the retinal microvasculature. Furthermore, pro-inflammatory cytokines such as IL-1β, TNF-α, IL-6, and IL-8 are significantly increased in diabetic patients and correlate positively with disease severity (60). Many of these cytokines attract neutrophils and induce NETosis/NET production. DR patients present with a high neutrophil-to-lymphocyte ratio, which correlates with disease severity, establishing its potential utility as a biomarker of DR severity (61). In addition, murine models of DR exhibit dense retinal neutrophilic infiltrates which adhere to endothelial cells, causing leukostasis (22). Circulating DNA-histone complexes and NE were significantly elevated in diabetic patients with retinopathy compared to those without retinopathy. These markers were significant independent risk factors of retinopathy (62, 63). In agreement with these findings, a later study demonstrated that injecting IL-8 or TNF-α intravitreally into the eyes of mice resulted in infiltration of neutrophils producing NETs, as evidenced by positive immunohistochemical staining for NE, MPO, and cit-H3. These markers were also elevated in vitreous samples of patients with PDR and, also, correlated significantly with its severity (64).

Taken together, these findings suggest that chronic hyperglycemia disrupts the inner blood-retinal barrier, resulting in non-proliferative diabetic retinopathy (65). This disruption also can result in neutrophil migration into the retina, causing leukostasis and inflammation, two events crucial for DR progression (65). Subsequently, the pro-inflammatory cytokines listed above may be responsible for NET production. Hyperglycemia itself also induces NETosis in a dose-dependent manner (22, 62). Regarding the specific contribution of NETs in DR pathogenesis, the NET component NE has been shown to contribute to vascular damage in DR (66). Another important neutrophil protein is lipocalin-2 (LCN2), which is elevated in cases of PDR and has been shown to activate HIF-1α (67–69). LCN2 may also inhibit the degradation of matrix metalloproteinase 9 (MMP9) to enhance apoptosis of retinal capillary cells and promote angiogenesis and neovascularization (70).

From a clinical standpoint, DNAse-1 abrogates NETs in the anterior and posterior chambers of mice eyes (64). Seeing as NE may contribute to the pathogenesis of DR, future research should assess if inhibiting this enzyme brings any benefits in ameliorating the severity of DR. LCN2 also warrants extensive analysis to fully uncover its use as a potential biomarker for DR severity and as a therapeutic target in this setting. In this regard, inhibiting serine/threonine kinase AKT2, an upstream regulator of LCN2, has been utilized in the early stages of age-related macular degeneration to reduce inflammation, neutrophil infiltration, and activation of retinal glial cells, but it has yet to be tested in models of DR (71).

Other than LCN2, numerous studies have shown the benefit of mitogen-activated protein kinase (MAPK) inhibitor annexin-1 in attenuating the microvascular complications of diabetes, namely nephropathy and cardiomyopathy, but their use in DR remains unknown (72). Another potentially useful class of such molecules is the lipidic pro-resolving mediators called lipoxins, specifically lipoxin-A4 (LXA4). LXA4 is synthesized in the inner retina and its levels decrease following eye injury (73). Both NPDR and PDR patients have lower serum levels of LXA4 compared to diabetics without retinopathy; lipoxin-A4 levels were also lower in the vitreous of PDR patients (74). LXA4 reduces the production of proinflammatory cytokines, including VEGF. Because this effect is deficient in DR, assessing the potential benefit of exogenous LXA4 administration in DR could prove fruitful (75).

Lastly, the use of purinergic receptor blockers in age-related eye disorders, including DR, is informed by the high amount of ATP released in these conditions, which bind to P2X_7_ receptors. Stimulation of P2X_7_ receptors positively regulates a HIF-1α-mediated expression of VEGF, thereby propagating angiogenesis and neovascularization in PDR (76). In this context, blocking the P2X_7_ receptor has been shown to reduce inflammation in rat models of DR (77). Two different P2X_7_ antagonists, A740003 and AZ10606120, have recently been shown to reverse increased vascular permeability and VEGF expression in a model of streptozotocin-induced hyperglycemic retinopathy in rats. The production of many cytokines, such as IL-1β and TNF-α, is also abolished by P2X_7_ inhibition. These cytokines, as discussed above, activate NETosis, suggesting that elimination of NETosis may be one of the pathways by which P2X_7_ antagonists exert their beneficial effects. The specific pathways activated by P2X_7_ and its utility as a therapy for DR were recently described in a review (76).

An excellent review by Martínez-Alberquilla et al. detailed the specific effects of these medications in age-related eye disorders, including DR (65). This paper also raised an important point with regards to NET markers: future comparative studies should evaluate whether NET markers in tear film and on the ocular surface are reliable predictors of DR compared to blood and intra-ocular samples, as they are much easier and less invasive to attain. The use of such techniques is suggested as a potential avenue to monitor ocular and neurodegenerative diseases, with LCN2 being suggested as a biomarker and therapeutic target in both conditions (78). Future comparative studies assessing blood, intra-ocular, and tear film NET markers as independent and combined predictors of DR severity and progression will undoubtedly shed some light on what is the most accurate sampling method to measure these biomarkers.

### Diabetic nephropathy

Diabetic nephropathy (DN) is a form of chronic kidney disease (CKD) that occurs in 20–40% of long-standing diabetics (79, 80). The progression of diabetes-associated CKD to end-stage renal disease (ESRD) is drastic, with 50% of T1DM patients developing ESRD within 10 years of onset of their kidney disease symptoms, specifically proteinuria. This makes DM the leading cause of ESRD globally (81). While chronic hyperglycemia is the key player in the pathogenesis of diabetic nephropathy, it alone does not explain all the pathologic changes which occur in this disease. In this context, recent evidence has considered inflammation crucial in the progression of diabetic nephropathy.

The pathophysiological hallmarks of diabetic nephropathy culminate in glomerular endothelial cell injury, resulting in the expression of adhesion molecules P- and E-selectin on endothelial cells that attract immune cells such as neutrophils. Furthermore, NLRP3 (NOD-, LRR- and pyrin domain-containing protein 3) inflammasome activation is recognized to underpin DN pathogenesis and proinflammatory cytokines such as IL-6, IL-8, TNF-α, IL-17 (a neutrophil chemoattractant), and IL-18 are significantly elevated in the serum and urine of diabetic patients (82). Neutrophil adhesion and homing are significantly increased in diabetic patients with overt proteinuria compared to diabetic normoalbuminuric patients and healthy, non-diabetic controls (83). Neutrophils in diabetics also show an activated phenotype with increased ROS production, which may damage endothelial cells to cause DN progression (84). Thus, the data suggest that neutrophil homing into DN kidneys may, at least in part, contribute to glomerular inflammation with subsequent scarring.

NETosis is a ROS-dependent process, and the inflammasome-dependent cytokines IL-1β and IL-18 are well-established inducers of NETs. Hyperglycemia itself, which is the major factor underpinning DN pathogenesis, also induces NETosis. There is also a wealth of data linking NETs to inflammatory kidney disorders, including acute kidney injury, systemic lupus erythematosus, and the ANCA-positive vasculitides (85–88). Judging by these observations, it can be assumed that NETs play a role in DN, albeit a direct link between NETs and diabetic nephropathy remains yet to be demonstrated. A study demonstrated significantly higher levels of circulating cell-free DNA in T2DM patients with nephropathy compared to those without nephropathy. Furthermore, T2DM patients in general showed higher levels of NET markers, including nucleosomes, dsDNA, and NE (89). Consistent with this study, Miyoshi et al. demonstrated that higher MPO-DNA levels in T2DM patients correlated positively with clinical and laboratory parameters regarded as risk factors (e.g., prolonged disease duration, elevated BMI, albuminuria, and elevated liver function tests) for microvascular complications including DN (90).

The same study showed that hyperglycemia induces NETosis *via* the polyol pathway (90), which has been supported by other studies (91). Accordingly, treatment with ranirestat—an aldose reductase inhibitor (ARI) acting upstream of the polyol pathway by inhibiting sorbitol production—eliminates NET formation (90). Contrastingly, Sotrastaurin, an inhibitor that acts more downstream in the polyol pathway, only partially mitigates NETosis (90). ARIs are used clinically for the treatment of diabetic neuropathy and retinopathy, but numerous studies have also tested their use in DN. For instance, Tolrestat was found to prevent glomerular hypertrophy, mesangial expansion, glomerular basement membrane thickening, mesangial expansion and hypocontractility, and progression of albuminuria in streptozotocin-induced diabetic rat models (92). Epalrestat, another ARI, significantly improved renal arterial blood flow, which plays a protective role in early-stage DN (93). Furthermore, it was recently demonstrated that epalrestat significantly reduced albuminuria, the fusion of podocyte foot processes, and interstitial fibrosis in the kidneys of *db/db* mice (a widely used mouse model of T2DM) (94). In agreement with these findings, treating microalbuminuric T2DM patients with epalrestat for 5 years prevented significant increases in urinary albumin excretion (95). These results suggest that inhibition of the polyol pathway is a potential strategy for the treatment of DN.

Seeing as inhibiting aldose reductase significantly abolishes NETosis, perhaps NET inhibition could account for one of the pathways by which ARIs achieve their therapeutic effect in diabetes. However, serum levels of NET markers may not be reflective of potential renal NET infiltration. NETs also have not yet been physically visualized in the kidneys of DN. In this regard, assessing for the presence of NETs by immunofluorescence and quantifying their presence on renal biopsy specimens would directly implicate NETs in DN pathogenesis, as has been done in the case of lupus nephritis (96). Therefore, questions for future research include: are NETs physically present in DN kidneys? Do NETs presence in DN kidneys or elevated circulating NET markers correlate with clinical and laboratory markers of DN severity? Does inhibiting NET formation or accelerating NET degradation clear NETs within kidneys? And does NET inhibition result in clinical improvement in renal function?

### Impaired wound healing

Diabetic foot ulcers (DFUs) are ulcers or wounds commonly located at the bottom of the foot that occur due to both atherosclerotic peripheral artery disease and peripheral neuropathy. DFUs are responsible for more diabetes-related hospitalizations than any other diabetic complication (97). But more importantly, 40% of patients presenting with the diabetic foot will require amputation, making diabetes mellitus the leading cause of non-traumatic amputations worldwide (98); Zhang et al. identified the prevalence of DFUs to be as high as 13% in North America (99).

Physiologic wound healing comprises 4 stages: hemostasis, inflammation, proliferation, and remodeling. Many conditions disrupt one or more of these steps to impair wound healing. These conditions include either local (e.g., ischemia, foreign bodies, infection) or systemic perturbations (e.g., DM, obesity, medications, hypothyroidism, etc.) (100). On a mechanistic level, many of these factors share common pathophysiology in that they propagate an inappropriate inflammatory response detrimental to wound healing.

As neutrophils are the first cells to be recruited to sites of acute inflammation, their contribution to physiologic and pathologic wound healing has garnered much interest. Mechanistically, neutrophilic migration into wounds may be mediated by factor XII (FXII) (101). Indeed, FXII-deficient mice show reduced neutrophil infiltration into wounds compared to WT mice. FXII is synthesized by neutrophils themselves and mediates the trafficking of neutrophils into sites of inflammation (102). This effect is mediated by FXII binding to urokinase plasminogen activator receptor (uPAR) that downstream induces expression of integrin. Importantly, activation of uPAR by FXII results in increased intracellular calcium and NETosis. FXII-deficient mice showed decreased inflammation and faster wound resolution than WT mice, consistent with a detrimental role of NETs in diabetic wound healing (102).

As mentioned above, hyperglycemia causes NET production, which predisposes diabetics to higher levels of NETosis and consequently impaired wound healing (27). NETs are abundant in mice wounds and are absent in unwounded skin (103). Proteomic analysis of non-healing vs. healing DFUs showed enrichment of NET-related proteins, including NE, histone H4, and neutrophil proteinase, in the non-healing group (104). A recent study demonstrated PAD4 overexpression at surgical sites after total joint arthroplasty in insulin-resistant subjects compared to insulin-sensitive subjects, which is associated with delayed surgical wound healing (105). Wounds in PAD4 knockout mice heal significantly faster than in WT PAD4-positive mice; on day 14 after the mice were subject to excisional skin wounds, 80% of all wounds in PAD4-depleted mice had healed, whereas only 25% healed in PAD4-positive mice (103). Yang et al. recruited a cohort of diabetic patients with active foot ulcers, calculated clinical scores of DFU severity, and evaluated for a potential correlation between NET-specific markers and the clinical severity of DFUs (106). Indeed, NET-specific markers were significantly in DFU patients than in diabetics without DFU or in healthy controls and correlated positively with diabetic ulcer severity score (DUSS) and the wound, ischemia, and foot infection (WIFI) DFU severity scores. Furthermore, DFU tissue NE levels were significantly higher in DFU cases that became infected and experienced delayed healing (106). This was supported by Fadini et al., who not only reported higher levels of NE but also proteinase-3 to positively correlate with the chances of wound infection (104). Lastly, cit-H3 was identified as an independent risk factor for impaired wound healing and amputation (106). Therefore, NE, proteinase-3, and cit-H3 may constitute biomarkers for the risk stratification of DFU patients.

To explain these findings, NE degrades the extracellular matrix (ECM) to delay wound healing (107). On the other hand, secretory leukocyte protease inhibitor (SLPI) degrades NE to counteract its pathologic effects. Accordingly, administering SLPI or other NE inhibitors accelerated wound healing (108, 109) while depleting SLPI has the reverse effect (110). MMPs in NETs also degrade the ECM. Chronic wounds indeed feature elevated protease levels (111). Tissue inhibitors of matrix metalloproteinase (TIMP) inhibit MMPs, but overproduction of MMPs in NETs overwhelms this mechanism; indeed, a higher MMP/TIMP ratio predicts poor wound healing (112). MPO stabilizes NETs in wounds and exerts pro-inflammatory effects *via* oxidative stress (113). Extracellular histones activate complement, endothelial cells, and platelets to endothelial cells to promote local inflammation and hypercoagulability, reducing blood flow to wound areas delaying clearance of dead tissue, and impairing wound healing (114–116). NET components interact with many of the cellular constituents of wounds, including endothelial cells, macrophages, keratinocytes, and fibroblasts, a topic which has been covered extensively by Zhu et al. (27).

Specifically in diabetic wounds, excessive NET production activates the NLRP3 inflammasome in macrophages to increase IL-1β production, which in turn induces NETosis. The impact of this positive feedback loop is underscored by the elimination of NETs, significantly benefiting wound healing by reducing NLRP3 levels and mitigating the development of a pro-inflammatory M1 macrophage phenotype (117). Hence, future studies should further explore the clinical benefit of targeting this pathway. In this regard, milk fat globular epidermal growth factor VIII (MFG-E8) attenuates the NLRP3-NET axis to promote inflammation resolution and wound healing (118). Consistently, MFG-E8-deficient mice display dense neutrophilic infiltration into wounds and subsequent excessive NET production, associated with delayed wound closure (118). Another protein, leucine-rich alpha-2-glycoprotein 1 (LRG-1), although essential for timely wound closure, is elevated in diabetic patients and mice, where it causes NETosis which, when excessive such as in diabetics, actually impairs normal wound healing. Indeed, depleting LRG1 in mice is protective against impaired wound healing, acting partly through dampening inflammation by reducing NET overproduction (119).

Protein Kinase C βII (PKC βII) is another mediator of NET production; administering a PKC βII inhibitor such as ruboxistaurin reduces NET production, increases local perfusion to wounds, and increases endothelial cell proliferation, all encouraging physiologic wound healing (120). Gonadotropin-releasing hormone (GnRH) can bind to GnRH receptors on neutrophils to induce NETosis to impair wound resolution while administering GnRH antagonists reduces NET formation and wound size (121). Like in normoglycemic wounds, PAD4 depletion or inhibition by Cl-amidine in diabetic wounds reduces NET markers and accelerates wound healing (103). Similar to the MAPK inhibitor annexin-1 which has shown benefit in preventing the microvascular complications of diabetes, investigators have utilized Na_2_S to inhibit MAPK, which dampens NETosis with a corresponding acceleration in wound healing (122). In summary, all these mediators constitute potential therapeutic targets, the inhibition of which could be of significant clinical benefit in the management of DFUs.

## Metformin and NETs

Current guidelines—as per the American Diabetes Association—recommend Metformin as the drug of choice for the initial management of T2DM patients at the time of diagnosis (123, 124). This recommendation is underpinned by various clinical trials showing Metformin to be an effective agent in lowering plasma glucose and HbA1C levels but without the risk of weight gain or severe hypoglycemia. Clinical trials have also demonstrated long-term metformin use to reduce the incidence of MACEs, cardiovascular mortality, and all-cause mortality in T2DM patients (125–127). Metformin may also reduce ischemia-reperfusion injury and thereby infarct size of MI in diabetic and non-diabetic mouse models (128).

However, although these beneficial clinical effects of Metformin have been intensively studied and characterized, the mechanisms of its effects remain investigational. The well-known glucose-lowering effect of Metformin is secondary to the suppression of hepatic gluconeogenesis and enhanced skeletal muscle glucose uptake (129). However, Metformin is also under investigation for use in treating cardiovascular disease irrespective of diabetes status, underscoring its yet-to-be understood glucose-independent mechanisms. This is further reinforced by the apparent beneficial effects of Metformin in aging, COVID-19, and various types of cancers, conditions unrelated to blood glucose changes (130).

In this context, Metformin reduces neutrophil-to-lymphocyte ratios in patients with diabetes (131). Furthermore, Metformin reduces levels of NETosis in neutrophils of T2DM patients (132). A subsequent study by Menegazzo et al. reported metformin treatment as reducing serum NE, proteinase-3, citrullinated histone, and dsDNA levels (133). Metformin blunted NETosis in neutrophils after exposure to classical NET-inducing stimuli, and this effect was independent of the anti-hyperglycemic effect of Metformin (133). The potential clinical significance of the inhibition of neutrophils and NETosis by Metformin has also been studied. Lipocalin—a NET protein discussed above in the context of DR—is found in carotid artery atheromas in diabetic patients, particularly in complicated plaques (134). In agreement with this, higher lipocalin levels in plaques were associated with an increased risk of cerebral embolization. Metformin significantly reduced leukocyte recruitment into carotid plaques of diabetes and decreased lipocalin levels (134). Lastly, a study on rat models reported that treatment with Metformin before MI significantly attenuates pathologic cardiac remodeling and fibrosis by mitigating neutrophil recruitment and NET production, evidenced by lower neutrophilic infiltration into the infarcted myocardium on histopathological examination and reduced MPO activity (135). In summary, Metformin has been shown to reduce the risk of adverse cardiovascular outcomes in diabetic patients *via* mechanisms unrelated to glycemic control and potentially involving the inhibition of NETosis.

Severe COVID-19 also features dysregulated neutrophil responses with increased NET production, contributing to the systemic inflammatory response syndrome and immunothrombosis in these patients (136, 137). DM patients are known to be predisposed to severe COVID-19. Given that NETs play essential roles in the immunologic and clinical phenotypes of severe COVID-19 and that many diabetic patients are prescribed Metformin, studies looked into the potential benefit of Metformin in people with diabetes who developed COVID-19. Metformin reduced in-hospital mortality of diabetic patients who contracted COVID-19 (138, 139). Cancer is another disease in which NETs are involved; NETs promote the acquisition of various hallmarks of carcinogenesis, including invasion, angiogenesis, immune evasion, and metastasis (140, 141). A recent study demonstrated high levels of mitochondrial NET formation in circulating neutrophils of hepatocellular carcinoma (HCC) patients, which promote the expression of metastasis-promoting inflammatory mediators. Metformin ameliorated mitochondrial NET production and mitigated the metastatic capacity of HCC cells (142). Therefore, the NET-targeting mechanism of Metformin broadens its clinical indication beyond just glycemic control in T2DM patients to conditions such as COVID-19 and cancer.

Regarding diabetes complications, numerous mechanistic studies show Metformin to reduce the development of DR (143) and accelerate wound healing (144, 145), but unfortunately, clinical data on the latter is limited (146). Therefore, observational studies comparing the incidence of impaired wound healing in diabetic patients receiving Metformin with controls are needed. Nevertheless, whether NET inhibition partially contributes to this beneficial effect remains unknown. Future investigations into this topic may rationalize novel drug combinations, whereby Metformin and other NET-targeting medications may synergize to benefit diabetic patients at risk of these complications.

## Discussion

In this review, we set out to summarize the important laboratory and clinical studies supporting the role of NETs in diabetic complications, in the hopes to inform the focus of future studies on this pertinent topic. However, many questions remain to be addressed, the answers to which will undoubtedly further the discussion surrounding NETs and diabetic complications.

Firstly, NET heterogeneity must be explored further; many different types of NETs exist, including the classic lytic NETs, vital NETs, and mitochondrial NETs. These NETs are produced under different circumstances secondary to distinct stimuli, have varying compositions, may have differential contributions to diabetic complications, and respond differently to anti-NET therapies. The focus of future research should therefore involve elucidating the relative contributions of each of these NETs in each complication. Classifying the different types of NETs at sites of pathology will also provide further insight into the probable NETs-inducing stimuli involved in these pathways. The potential compositional variations between different NETs types may underpin variable clinical responses to NET-targeting medications. Another factor to consider in diabetic complications is the effect of age on the propensity of neutrophils to produce NETs. Indeed, a recent study by Lu et al. showed aged neutrophils to produce more NETs than younger ones (26). Given that age is a major risk factor in the development of diabetes and its complications, perhaps one of the many effects of aging is reflected in neutrophils producing more NETs. Intriguingly, this study found major gender-specific differences in neutrophil gene expression (26). In the context of NETs heterogeneity, it would be interesting if future research decides to explore the composition of NETs which are elaborated by aged neutrophils and any potential sex-specific differences. The study by Lu et al. examined primary bone marrow neutrophils, paving the way for studies evaluating these relationships in blood circulating neutrophils.

Secondly, while we elaborate on numerous ways to inhibit NETs, such as accelerating NET degradation, inhibiting certain NET components, or inhibiting NET production, diabetic patients are immunosuppressed and susceptible to various debilitating infections. Inhibiting an integral neutrophil function could exacerbate this condition. Therefore, the indication and side-effect profile of NET-inhibiting drugs should be clearly defined (which will require large-scale clinical trials), and more local routes of administration, such as topical applications for DFUs or eyedrops for DR, be further explored. Related to this point, it is also important to consider that NETs are at the end of the day a physiologic neutrophil response aimed at protecting the body, and many of its beneficial effects have been identified. Therefore, when exactly do NETs switch from being physiologic to pathologic is an interesting topic for future work to address.

Thirdly, investigators must continue to describe the precise mechanism by which NET components—such as histones, extracellular DNA, and proteases such as MMPs—contribute to diabetes complications. A reason for this is that histones, DNA, and MMPs are not always present in tissues as NET components; for instance, DNA and histones are released during necrosis of tissue-resident cells. Furthermore, whether DNA-bound or free histones exert different local effects remains unclear. Cellular senescence is a hot topic that reportedly plays a role in the pathogenesis of various age-related diseases, including diabetes. In the context of wound healing, transient senescence is critical to physiologic wound healing, but chronic senescence conversely impairs wound healing (147). In diabetic wounds, levels of senescent cells are higher, and senescent macrophages secrete abundant C-X-C motif chemokine receptor 2 (CXCR2) ligands as part of secretory phenotype (SASP) (148, 149). Since neutrophil homing into diabetic wounds with subsequent NET production has been demonstrated, and given that CXCR2 is well-recognized as a neutrophil chemokine receptor, an interplay between elevated senescent cell burden and NET production may be reasonable to investigate. Additionally, senescent cells release MMPs which degrades the ECM to drive age-related loss of skin elasticity (150). As described above, NET-derived MMPs also contribute to impaired wound healing. Therefore, dissecting the differential contributions of senescence and NET-derived MMPs and their relative importance to chronic diabetic wounds may inform future strategies to target certain mechanisms over others to improve wound healing in diabetics.

Lastly, no drug specifically targeting NETs is currently approved for clinical use. In Figure 1 we describe different NET-targeting strategies, which preclinical and clinical data have substantitated in the context of diabetes. However, it should be noted that DNAse-1—despite its apparent benefits in accelerating the breakdown of coronary and cerebral thrombi—has in cases been harmful to bronchiectasis patients in one clinical trial (151). The mechanism underpinning may involve the release of DNA-bound NE and other proteases into tissues and circulation, where they aggravate tissue injury; DNase treatment can even increase NE activity (152). Nevertheless, randomized clinical trials showed that DNase-1 administration significantly improved oxygen saturation and lung compliance in severe COVID-19 pneumonia patients compared to controls who did not receive DNase-1 (153). This was presumably through degrading lung NETs, as reductions in BALF MPO levels were noted in treated patients (153). This further underscores the point of specifying the clinical indications of NET-targeting drugs, which necessarily will require the outcomes of well-designed, robust clinical trials. Therefore, current focus should be on translating important benchwork into clinical data. An interesting recent study revealed disulfiram to ameliorate NETs to ameliorate the pathology of transfusion-related acute lung injury in mice and also COVID-19 lung injury in golden Hamsters (154). Therefore, testing disulfiram in other NET-related disorders, including diabetes complications, should be explored by future studies. But again, the heterogeneity of NETs in diabetic complications—in terms of composition and stimuli—remains unexplored. Studying the intrinsic mechanisms behind NETosis and potential inducers in this context will not only possibly reveal novel therapeutic targets but also better specify the precise clinical indications of already existing drugs that could be repurposed to prevent or treat diabetic complications *via* their NET-targeting mechanism.

In summary, we believe NETs are potentially significant executors in the pathogenesis of diabetic macrovascular and microvascular complications. Future research expectedly will continue to expand upon the association between NETs and major complications to rationalize clinical practice-changing trials that will benefit diabetic patients and the healthcare system.

## Author contributions

AS and AY: conceptualization. AS, SAb, OA, and SAl: writing—original draft preparation. AS, AA, JK, KA, and AY: writing—review and editing. JK, KA, and AY: supervision. All authors have read and agreed to the published version of the manuscript.

## Conflict of interest

The authors declare that the research was conducted in the absence of any commercial or financial relationships that could be construed as a potential conflict of interest.

## Publisher's note

All claims expressed in this article are solely those of the authors and do not necessarily represent those of their affiliated organizations, or those of the publisher, the editors and the reviewers. Any product that may be evaluated in this article, or claim that may be made by its manufacturer, is not guaranteed or endorsed by the publisher.
